# Rapid Identification of Candidate Genes for Seed Weight Using the SLAF-Seq Method in *Brassica napus*

**DOI:** 10.1371/journal.pone.0147580

**Published:** 2016-01-29

**Authors:** Xinxin Geng, Chenghong Jiang, Jie Yang, Lijun Wang, Xiaoming Wu, Wenhui Wei

**Affiliations:** Oil Crops Research Institute of the Chinese Academy of Agricultural Sciences/Key Laboratory of Biology and Genetic Improvement of Oil Crops, Ministry of Agriculture, Wuhan, 430062, China; Huazhong university of Science and Technology, CHINA

## Abstract

Seed weight is a critical and direct trait for oilseed crop seed yield. Understanding its genetic mechanism is of great importance for yield improvement in *Brassica napus* breeding. Two hundred and fifty doubled haploid lines derived by microspore culture were developed from a cross between a large-seed line G-42 and a small-seed line 7–9. According to the 1000-seed weight (TSW) data, the individual DNA of the heaviest 46 lines and the lightest 47 lines were respectively selected to establish two bulked DNA pools. A new high-throughput sequencing technology, Specific Locus Amplified Fragment Sequencing (SLAF-seq), was used to identify candidate genes of TSW in association analysis combined with bulked segregant analysis (BSA). A total of 1,933 high quality polymorphic SLAF markers were developed and 4 associated markers of TSW were procured. A hot region of ~0.58 Mb at nucleotides 25,401,885–25,985,931 on ChrA09 containing 91 candidate genes was identified as tightly associated with the TSW trait. From annotation information, four genes (GSBRNA2T00037136001, GSBRNA2T00037157001, GSBRNA2T00037129001 and GSBRNA2T00069389001) might be interesting candidate genes that are highly related to seed weight.

## Introduction

*Brassica napus* (*B*. *napus*) is one of the most important oil crops and also the third largest oilseed crop worldwide. It supplies more than 13% of the world's vegetable oil and is a major economic crop [1]. Breeding of high yield oilseed crops is always the target and primary mission of plant breeders. Seed weight (SW), siliques per plant (SPP) and seeds per silique (SPS) are three important and basic components to determine the seed yield per plant [2]. Seed weight is the most important component and is a direct trait for yield of oilseed crops. To increase the seed weight is a major approach to improve the yield of oilseed crops [3]. Therefore, understanding the genetic determinants of seed weight is of great significance for yield improvement in oilseed breeding [4].

Exploring new quantitative trait loci (QTLs) for seed weight with molecular-marker-assisted selection is always a hot topic to improve *B*. *napus* seed yield [5]. To date, several QTLs related to seed yield have been identified and functionally characterized [2, 3, 6, 7]. In addition, more and more QTLs for seed weight have been detected and mapped on all the 19 chromosomes of *B*. *napus* [3, 4, 8–12]. The genetic basis of seed weight is complicated and also related to oil and protein content [11]. Presently, the genetic mechanism for this important quantitative trait is still not clear and no gene which regulates seed weight has been fine mapped or cloned due to the complicated genomic structures and unavailable genome sequence information for *B*. *napus* before [7, 13]. However, the relevant *B*. *napus* genome sequence information has been published recently, which provides a rich bioinformatics research platform for studying the genetic mechanism of seed weight in our research.

Specific-locus amplified fragment sequencing (SLAF-seq) is a kind of highly efficient method for large-scale genotyping, which combines an enhanced reduced representation library (RRL) technology and high-throughput sequencing methods to discover SLAF markers (including SNP and Indel markers) and genotype large populations or bulked segregant [14]. SLAF-seq has emerged as a highly automated, reduced and high-resolution method to develop specific molecular markers. It has several positive characteristics such as high efficiency for marker development, low cost, less sequencing demand and high capacity for large populations, which has allowed SLAF-seq to become widely used for large-scale marker discovery, high-density genetic map development, hot-spot region association with important trait identification and etc. [5, 14–18].

The SLAF-seq technology has been successful in developing 89 specific molecular markers and creating a genetic map for *Thinopyrum elongatum* and common carp with high quality SLAFs [15]. Sun et al. [14] conducted a pilot study on rice and soybeans and selected 21,000 and 76,000 SLAFs by *HaeIII* and *MseI* digestion, respectively. Li et al. [18] constructed a high-density genetic map based on large-scale markers developed by SLAF-seq and applied these markers to QTL analysis for isoflavone content in *Glycine max*. Xia et al. [17] identified 56,635 SLAF tags and three trait-related candidate regions on Chr3 in maize with 51 candidate genes and a size of 3.947 Mb by SLAF-seq technology. Qi et al. [16] constructed a high-density genetic map for soybeans based on SLAF-seq. Xu et al. [5] selected 40,114 SLAFs after screening low quality SLAFs for further analysis and found two marker-intensive regions at 24,600,000–24,850,000 bp and 25,000,000–25,350,000 bp on chromosome 3 which were identified to be tightly associated with the 1000-grain weight in rice by SLAF-seq technology. Recently, SLAF-Seq has been successfully and widely used to obtain sufficient markers from whole genomes to construct high-density genetic maps for sesame and soybeans [16, 18, 19]. Association analysis to identify hot-regions associated with important traits for maize [17] and rapid identification of major QTLs associated with rice grain weight have also been performed [5]. All of these studies have provided strong evidence for the application of SLAF-seq technology.

Bulked-segregant analysis (BSA) is a traditional method to identify DNA markers tightly linked to target gene (s) for a given phenotype. Combining BSA and SLAF-seq technologies has been successfully proven to be an efficient way for candidate gene identification in plants [17]. In this study, SLAF-seq technology was first used to identify candidate genes of TSW by sequencing two bulked segregate DNA samples and parental DNA samples based on the genomic sequence of *B*. *napus*. Then, four associated markers for seed weight were obtained and a hot region ~0.58 Mb at 25,401,885–25,985,931 bp with 91 candidate genes on ChrA09 was identified to be tightly associated with the TSW trait. From annotation information, four interesting candidate genes, GSBRNA2T00037136001, GSBRNA2T00037157001, GSBRNA2T00037129001 and GSBRNA2T00069389001, which participate in seed development, cell division and IAA biosynthetic processes, might be highly related to seed weight.

## Materials and Methods

### Plant materials

A DH population with 250 lines was derived from a cross between two parents, a large-seed line G-42 and a small-seed line 7–9, through microspore culture and doubling technology [20]. All 250 DH lines along with both parent plants were grown in the field under standard conditions from October 2013 to May 2014 at the experimental farm of the Oil Crops Research Institute of the Chinese Academy of Agricultural Sciences, Wuhan, China.

### Phenotypic observation

**Fig 1:**
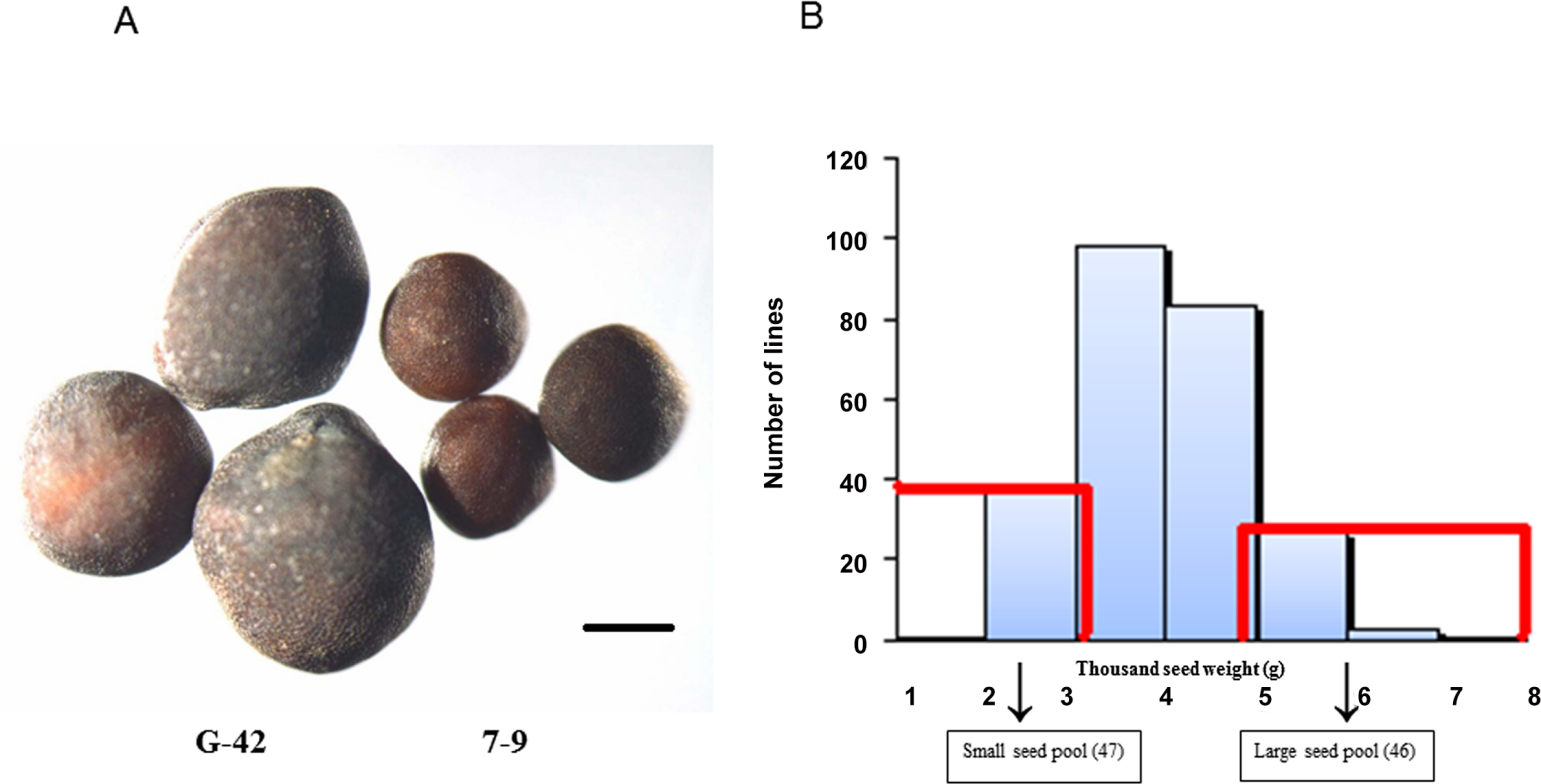
The seed phenotype of two parent lines and two extreme pools were selected by 1000-seed weight (TSW) data for SLAF-sequencing. (A) Seeds of the large-seed line G-42 and the small-seed line 7–9. (Scale bar, 1 mm) (B) Forty-six lines with the heaviest TSW and 47 lines with the lightest TSW were selected and pooled for SLAF-sequencing (the histogram was drawn based on the TSW data collected from 250 DH lines in May, 2014).

Three plants per line of the DH population and two parent plants (Fig 1A) were bagged at flowering time for harvesting pure seeds. After harvesting and drying, the fully dried seeds were collected to measure seed weight trait. The TSW was evaluated from the weight for 1000 seeds and the mean values of 1000-seed weights for three replicates of each line in this experiment (S1 Table).

### Two extreme DNA bulks construction

#### Two segregating pools selection

Two DNA bulks for sequencing were first made by selecting extreme individuals from the 250 DH population plants with the basic statistics of the phenotypic data. The lightest 47 lines (G1-G47) were selected as the small-seed pool, and the heaviest 46 lines (G51-G96) were selected as the large-seed pool from 250 DH lines (Fig 1B, S1 Table).

#### Genomic DNA extraction

Total genomic DNA was isolated from young healthy leaves of two parents and the selected 93 DH lines using the cetyltrimethylammonium bromide (CTAB) method with some modifications and then purified by RNase [21]. DNA concentration and quality were estimated with a Nanodrop 2000 UV–Vis spectrophotometer (NanoDrop, Wilmington, DE, USA), and adjustments were made to yield a final DNA concentration of 100 ng. μl ^-1^ with a total DNA amount greater than 20 μg. The 46 individual genomic DNA of the large-seed group were equally mixed as a large-seed DNA bulk and meanwhile 47 individual genomic DNA of the small-seed group were equally mixed as a small-seed DNA bulk. Genomic DNA of two DNA bulks and both parents were prepared for following SLAF sequencing.

### SLAF library construction

A pilot SLAF experiment was designed to determine conditions and appropriate restriction enzymes for digestion that optimize SLAF yield and maximize SLAF-seq efficiency. Then, the SLAF library was constructed based on the result of the pilot experiment for SLAF selection. The procedure was followed by Sun et al. [14] with minor modifications. We used the reference genome of *B*. *napus*, which has a size of 1.2 Gb (download link: http://www.genoscope.cns.fr/brassicanapus/data/ [22]). Purified genomic DNA was digested into fragments of 314~344 bp in size with an appropriate restriction enzyme combination, *HaeIII*+*RsaI* (NEB, Ipswich, MA, USA). Subsequently, fragment ends reparation, index paired-end adapters’ ligation and adapter—modified ends obtainment were performed step by step. We selected the objective size on a 2% agarose gel and amplified the fragments through PCR reaction. Finally, we executed high-throughput sequencing by Illumina HiseqTM 2500 (Illumina, Inc; San Diego, CA, USA) at Biomarker Technologies Corporation in Beijing. Real-time monitoring was performed for each cycle during sequencing and the ratio of high quality reads with quality scores greater than Q30 (indicates a quality score of 30, indicating a 0.1% chance of an error and thus 99.9% confidence) in the raw reads and guanine-cytosine (GC) content was calculated for quality control.

### SLAF-seq data clustering, polymorphic analysis and associated markers identification

Dual-index software [23] was used to identify the SLAF-seq raw data and obtain the reads of each sample. Being digested by the same restriction enzyme, all SLAF pair-end reads of samples were clustered according to sequence similarity by the BLAF software [24]. Sequences with over 90% identity were clustered in one SLAF locus (or SLAF tag) and a large number of specific fragments were selected for specific molecular marker development. SLAF tags were developed and compared among different samples. Polymorphic SLAF tags showed polymorphism between the parents including two kinds of markers, SNP and Indel [25]. For the polymorphic screening, there were three kinds of SLAF tags: polymorphic SLAFs, no polymorphic SLAFs and repetitive SLAFs. Clusters with more than four tags were regarded as repetitive SLAFs and were filtered out. SLAFs with two, three, or four tags were considered to be polymorphic SLAFs and those with only one tag were considered to be no polymorphic SLAFs. In this study, polymorphic SLAFs with sequence depth of both parents less than 5X were defined as low-depth SLAFs and filtered out. Finally, the potential SLAFs with one genotype derived from the male parent (G-42) and the other from the female parent (7–9) were identified as SLAF markers, and were selected for further association analysis.

### Association analysis

The relative marker abundance in bulked DNA pool 1 (the small-seed pool) was calculated as the number of reads of the maternal allele divided by the number of reads of the paternal allele, whereas in pool 2 (the large-seed pool), the relative marker abundance was calculated as the number of reads of the paternal allele divided by those of the maternal allele. It was expected that the larger the relative abundance, the greater the possibility that the marker was associated with TSW. SNP-index association analysis [26] and Euclidean distance association analysis [27] were used in this research.

In this study, P stands for the male parent (G-42), M stands for the female parent (7–9), aa represents the small-seed pool and ab represents the large-seed pool.

#### SNP-index association analysis

SNP_index association analysis was recently published and is a type of method used to calculate genotype frequency differences between two bulks that are satisfied by Δ (SNP_index). The closer marker is associated with trait while the closer Δ (SNP_index) is associated with 1.

Δ (SNP_index) was calculated as follows: Maa is the depth of the aa group derived from M while Paa indicates the depth of the aa group derived from P; Mab means the depth of the ab group derived from M while Pab stands for the depth of the ab group derived from P.

SNP_index(ab)=Mab/(Pab+Mab);

SNP_index(aa)=Maa/(Paa+Maa);

Δ(SNP_index)=SNP_index(aa)-SNP_index(ab).

#### Euclidean distance association analysis

Euclidean distance (ED) association analysis is a type of method that calculates Euclidean distance (quadratic sum root of differences between bulks from the depth of four types of base) and is satisfied by ED. In theory, the higher the ED value is, the closer the object site.

ED was calculated as follows: A_aa_, C_aa_, T_aa_, and G_aa_ respectively represent the depth of bases A, C, T and G on a site in the large seed bulk. A_ab_, C_ab_, T_ab_, and G_ab_ represent the depth of bases A, C, T and G on a site in the small seed bulk, respectively.

$$ED=\sqrt{{\left(A_{aa}-A_{ab}\right)}^{2}+{\left(T_{aa}-T_{ab}\right)}^{2}+{\left(G_{aa}-G_{ab}\right)}^{2}+{\left(C_{aa}-C_{ab}\right)}^{2}}$$

In this study, we used SLAF-seq technology combined with BSA to detect polymorphic tags between the two bulked DNA pools and quickly identified marker intensive hot-regions for seed weight on the genome of *B*. *napus*.

## Results and Discussion

### SLAFs development

After SLAF library construction and high-throughput sequencing, a total of 24.18 M reads were developed to procure SLAFs (Table 1). The Q30 ratio was 88.18% and the GC content was 43.68% (Table 1). Of these high-quality data, 3,380,481 reads were from the male parent and 4,134,256 reads were from the female parent. Read numbers for the small-seed pool and small-seed pool were 9,453,088 and 7,216,711, respectively.

**Table 1:** Statistic results of sequencing data for both parents and bulked DNA pools.

Sample	Sample ID	Read number	Q30^a^ percentage (%)	GC percentage (%)
**Male parent**	P	3,380,481	88.63	42.01
**Female parent**	M	4,134,256	88.94	44.89
**Small-seed pool**	aa	9,453,088	87.25	45.47
**Large-seed pool**	ab	7,216,711	87.89	42.36
**Total**		24,184,536	88.18	43.68

^a^ Q30 indicates a quality score of 30, indicating a 0.1% chance of error and thus 99.9% confidence

The numbers of SLAFs in the male and female parents were 86,429 and 95,008, respectively. The total depth and average depth of male and female parents was 1,801,757 (18.96x) and 2,132,208 (24.67x), respectively. For the two bulked pools, the numbers of SLAFs in the small-seed pool and the large-seed pool were 90,719 and 111,205, respectively. The total depth and average depth of the small-seed bulk and the large-seed bulk was 4,752,291 (52.38x) and 3,640,746 (32.74x), respectively (Table 2). Totally, we ultimately selected 112,292 SLAFs for further analysis.

**Table 2:** Statistic results of SLAF tags for both parents and bulked DNA pools.

Sample	Sample ID	SLAF number	Total depth	Average depth
**Male parent**	P	95,008	1,801,757	18.96x
**Female parent**	M	86,429	2,132,208	24.67x
**Small-seed pool**	aa	90,719	4,752,291	52.38x
**Large-seed pool**	ab	111,205	3,640,746	32.74x

Among the 112,292 SLAFs that were detected in total, 7,536 SLAFs showed polymorphism between the two parents with a polymorphism rate of 6.71% (Table 3). The number of non-polymorphic and repetitive SLAFs was 104,270 and 486, respectively.

**Table 3:** Statistical results for each SLAF type.

Type	Polymorphic SLAF	No polymorphic SLAF	Repetitive SLAF	Total
**Number**	7,536	104,270	486	112,292
**Percentage (%)**	6.71	92.86	0.43	100

**Fig 2:**
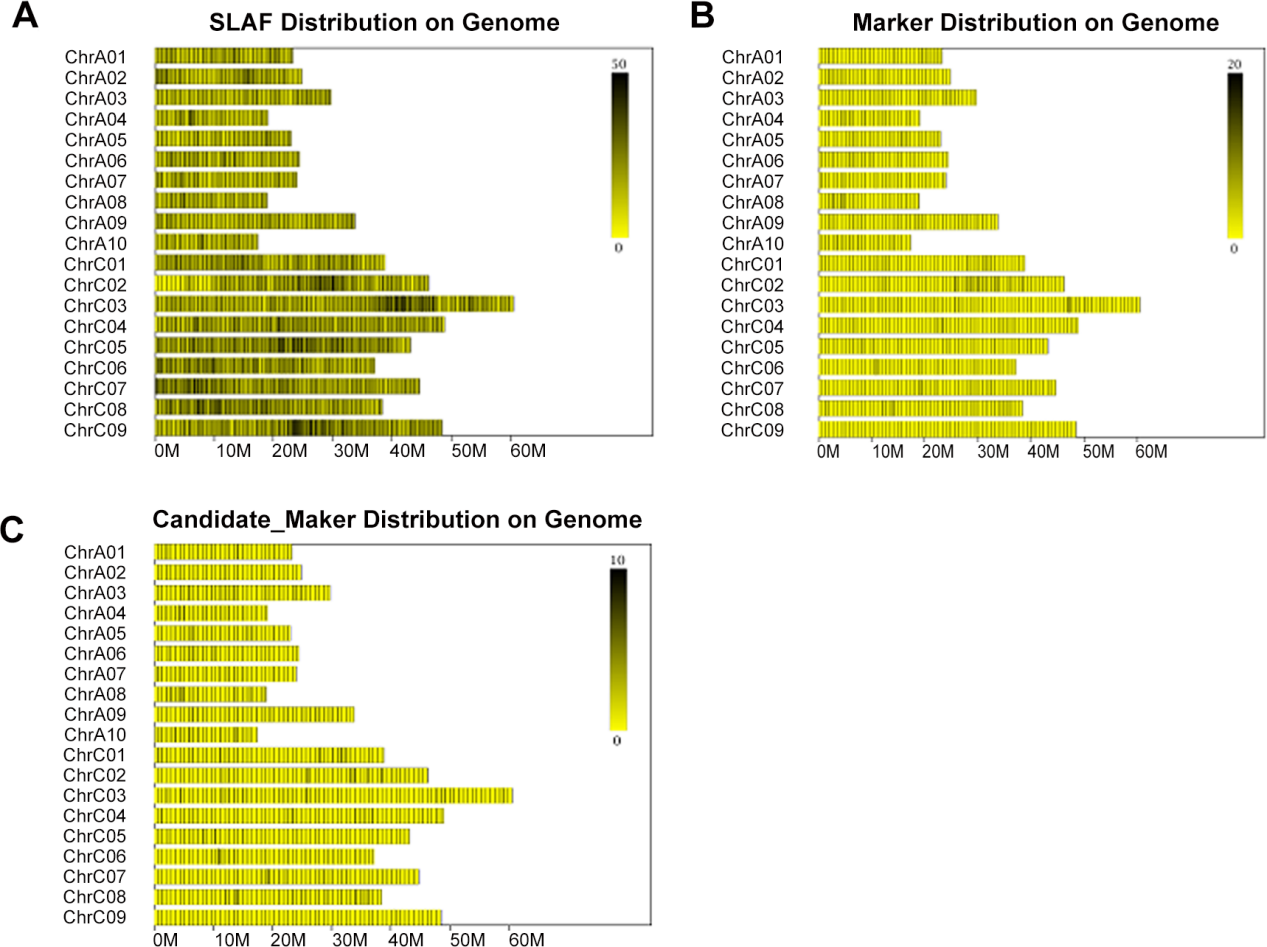
Distribution diagrams of all SLAFs, polymorphic SLAFs and candidate SLAF markers on the *B*. *napus* genome. (A) All SLAFs (black lines) distributed on each chromosome. (B) Polymorphic SLAFs (black lines) distributed on each chromosome. (C) Candidate SLAF markers (black lines) distributed on each chromosome. In each chromosome, the more the SLAF tags are, the darker the color is.

SLAF tags were located on the reference *B*. *napus* genome through short oligonucleotide analysis package (SOAP) software [28]. Statistics of marker numbers on each chromosome according to the positioning result were shown in Table 4 and a distribution diagram of SLAF on each chromosome was shown in Fig 2A. The SLAF tags were distributed equally on each chromosome.

**Table 4:** Number of all SLAFs, polymorphic SLAFs and high quality polymorphic SLAF number on each chromosome.

Chromosome ID	All SLAF number	Polymorphic SLAF number	High quality polymorphic SLAF number
**ChrA01**	3,542	269	60
**ChrA02**	3,722	282	67
**ChrA03**	5,079	445	142
**ChrA04**	2,624	324	112
**ChrA05**	3,162	346	99
**ChrA06**	3,613	334	100
**ChrA07**	3,299	327	103
**ChrA08**	2,902	281	98
**ChrA09**	5,085	492	136
**ChrA10**	2,602	350	117
**ChrC01**	6,707	420	118
**ChrC02**	7,324	596	108
**ChrC03**	11,201	629	164
**ChrC04**	8,554	430	77
**ChrC05**	9,297	410	86
**ChrC06**	6,930	368	102
**ChrC07**	8,674	486	117
**ChrC08**	8,022	460	114
**ChrC09**	9,953	287	13
**Total**	112,292	7,536	1,933

SLAF-seq is a newly developed, efficient and high-resolution strategy for large-scale *de novo* SNP and Indel markers discovery and genotyping of large population and bulked segregant [14] through sequencing the paired-ends of the sequence-specific restriction fragment length [16]. It has several advantages such as high efficiency for marker development, low cost, short cycle, high accuracy with less sequencing and a high capacity for large populations [14]. Compared with other inefficient, expensive, and time-consuming conventional methods for developing markers, such as next-generation sequencing, restriction-site associated DNA (RAD) sequencing, bar-coded multiplexed sequencing and etc.[29–31], SLAF-seq can develop large amounts of sequence information, enable its sequencing data to generate molecular markers directly, guarantee the efficiency, uniformity, quality and quantity of maker development and cover the whole genome [16]. Since the SLAF-seq methods were developed, they have been used in several studies, such as molecular markers development, major QTLs identification, candidate genes association analysis, high-density genetic mapping and etc..

In this study, we are the first to used SLAF-seq technology in *B*. *napus* combined with BSA to detect polymorphic markers between the two bulked DNA pools and parents. A total of 111,205 SLAF tags were developed as the basis for high-throughput sequencing and 7,536 polymorphic markers were identified between two parents. Finally, 1,933 high quality polymorphic SLAF markers were finally selected for further association analysis with quantity and quality meeting the requirements. The SLAF markers were well-distributed on each chromosome, and both the integrity and accuracy were very high (Fig 2B). In our study, we quickly identified a marker intensive hot-region for seed weight on ChrA09 through SLAF-seq technology combined with BSA. This method quickly detected major QTLs at a genome-wide level and delimited it to a narrower region.

### Polymorphic SLAF markers screening

A total of 7,536 polymorphic SLAFs were selected to obtain high quality polymorphic SLAFs after two rounds of sequencing and exclusion of low-quality fragments (Table 1). Tags with a depth less than 5X were excluded first. Then, with the reference genome sequence, potential SLAF tags with one genotype deriving from P and the other from M were identified as SLAF markers. Finally, 1,933 high-quality polymorphic SLAFs were selected as candidate SLAF markers for further association analysis. Statistics for high quality polymorphic SLAF marker numbers on each chromosome were shown in Table 4 and a distribution diagram of candidate markers on each chromosome was shown in Fig 2C.

### SNP_index association analysis

**Fig 3:**
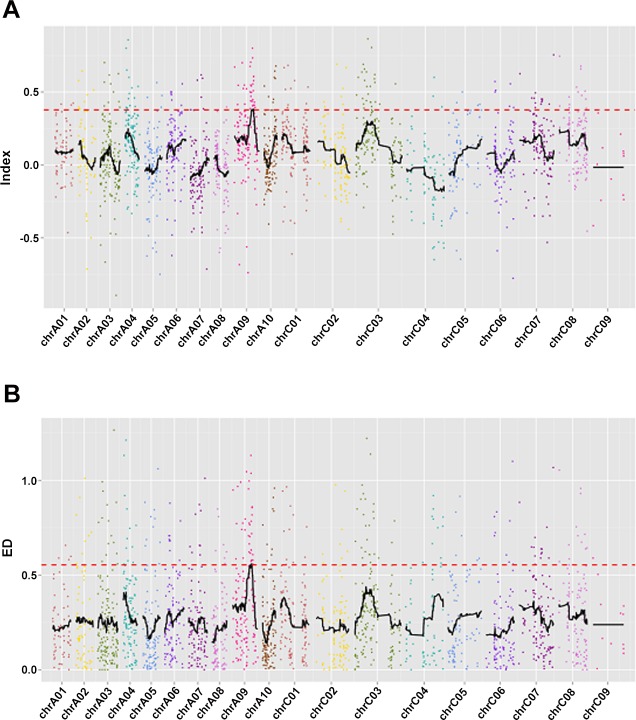
Identification of the hot-region for 1000-seed weight through two types of association analysis methods. (A) The results of SNP_index association analysis. The black lines show all fitting results of Δ (SNP_index), the red lines show the threshold of Δ (SNP_index). The larger the result of Δ (SNP_index) is, the stronger the association is. The association threshold was 0.3764 and 4 SLAF markers on ChrA09 significantly correlated with the seed weight trait. (B) The results of Euclidean distance association analysis. The black lines show all fitting results of ED, the red lines show the threshold of ED. The larger the result of ED is, the stronger the association is. The association threshold was 0.5532 and 4 SLAF markers on ChrA09 significantly correlated with the seed weight trait.

A total of 1,933 candidate polymorphic SLAFs were used for association analysis through the SNP_index method. The association threshold was 0.3764 and 4 SLAF markers on ChrA09 significantly correlated with the seed weight trait. The result of the SNP_index association analysis was shown in Fig 3A. Statistics for the number of associated SLAF markers on the chromosome were shown in Table 5. Through analysis of the 4 associated SLAF markers, a trait related candidate region on ChrA09 was identified. The candidate regions had a size of 0.58 Mb at nucleotides 25,401,885–25,985,931 with approximately 91 candidate genes in the region. The result of the candidate region identification by the SNP_index method was shown in Table 6.

**Table 5:** Number distribution of association markers on the chromosome by the SNP_index, Euclidean distance and Euclidean distance combined SNP index association analysis methods.

Association analysis methods	Chromosome ID	Association markers	Percentage (%)
**SNP_index**	ChrA09	4	100
**Euclidean distance**	ChrA09	4	100
**Euclidean distance combined SNP_index**	ChrA09	4	100
**Total**	ChrA09	4	100

**Table 6:** Information on the association region by the SNP_index, Euclidean distance and Euclidean distance combined SNP index association analysis methods.

Association analysis methods	Chromosome ID	Start	End	Size (Mb)	Associated marker number	Gene number
**SNP_index**	ChrA09	25,401,885	25,985,931	1	4	91
**Euclidean distance**	ChrA09	25,401,885	25,985,931	1	4	91
**Euclidean distance combined SNP_index**	ChrA09	25,401,885	25,985,931	1	4	91

### Euclidean distance association analysis

A total of 1,933 candidate polymorphic SLAFs were also used for association analysis through the Euclidean distance method. The association threshold was 0.5532 and 4 SLAF markers on ChrA09 were significantly correlated with the seed weight trait. The result of the Euclidean distance association analysis was shown in Fig 3B. Statistics for the number of associated SLAF markers on the chromosome were shown in Table 6. Through analysis of the 4 associated SLAF markers, a trait related candidate region on ChrA09 was identified. The candidate regions had a size of 0.58 Mb at nucleotides 25,401,885–25,985,931 with approximately 91 candidate genes in this region. The result of candidate region identification by the Euclidean distance method was shown in Table 6.

### Euclidean distance combined SNP_index association analysis

Euclidean distance and the SNP_index combined method were used for association analysis of 1,933 candidate polymorphic SLAFs. Four SLAF markers on ChrA09 were significantly correlated with the seed weight trait. The statistics of the number of associated SLAFs on the chromosome, the candidate regions and genes were shown in Tables 5 and 6. From all the results of three types of association analysis (Tables 5 and 6), we could conclude that the seed weight trait related candidate regions were at the same place.

In summary, it was shown that the candidate genes of seed weight were all located on ChrA09. It might verify the accuracy of SLAF-Seq through comparing with a linkage map of ChrA09 or the major QTLs of seed weight on ChrA09 in *B*. *napus*. We previously constructed a linkage genetic map using a F_2_ population derived from the same cross between rapeseed lines G-42 and 7–9 with 128 SSR markers and 100 SRAP markers and detected two major QTLs for SW. Two QTLs (QSW-X-A9-1 and qSW-W-A9-3) were both localized to ChrA09 [32]. They were located between two markers, Na14-B03 and CB10373-2, which were quite close to our candidate hot-region (25,401,885–25,985,931 bp) identified from SLAF-seq by blasting with *B*. *napus* reference genome [22]. In conclusion, compared to our previous QTL mapping, the candidate gene hot-region by SLAF-seq might be confirmed. To further validate the accuracy of these four associated SLAF markers, we chose 10 SLAF loci derived from 4 SLAF markers in 2 parents and 10 random individuals and performed independent traditional Sanger sequencing. Of these 120 genotypes, 117 were consistent and 3 were incorrect with the SLAF-seq genotyping information. Details are shown in S2 Table. The results compared by two types of sequencing ways confirmed the genotyping accuracy of SLAF-seq.

To deeply understand the differences and new findings in our research compared with other studies, we enumerated some similar work on *B*. *napus* seed weight QTLs. Li et al. [33] detected an association signals (position at 34, 653 kb) for seed weight on ChrA09 using association mapping which were consistent with some previous studies of quantitative trait loci mapping in *B*. *napus*. Li et al. [34] harbored two QTLs (their confidence intervals were on the position 30.68 Mb to 31.19 Mb for uq.A09-1and 29.02 Mb to 30.28 Mb for uq.A09-3) for both seed weight and silique length on ChrA09 by regional association analysis with a panel of 576 inbred lines in *B*. *napus*. Liu et al. [35] identified a major QTL on ChrA09 for both seed weight and silique length, which was confirmed to be the same one with Li et al. [34]. By fine mapping and association analysis, they finally uncovered a 165-bp deletion in the auxin-response factor 18 (*ARF18*) gene associated with increased SW and SL. Apparently, these QTLs or gene above for seed weight were totally different with ours. It is very likely that seed weight is quantitatively inherited, which is controlled by multiple QTLs [36].

### Association regional gene annotation

Totally, we obtained 4 polymorphic SLAF markers which narrowed the candidate associated regions down into 0.58 Mb in size on ChrA09, with a total of 91 genes. Ninety-one candidate genes were blasted with Gene ontology (GO) [37], Cluster of Orthologous Groups of proteins (COG) [38], Kyoto Encyclopedia of Genes and Genomes (KEGG) [39], Swiss-Prot [40] and Non-redundant protein (NR) [41] databases by BLAST software [42] yielding 90 genes that were successfully annotated. All the annotated information was listed in Table 7 and S2 Table. Of these candidate genes, 90 could be annotated in NR database; 70 could be involved in Swiss-Prot database; 87 could be included in GO database; 25 could participate in KEGG pathway and 35 could find annotated information in COG database.

**Table 7:** Statistics of association regional gene annotation.

Annotated databases	Annotated gene number
**NR**	90
**Swiss-Prot**	70
**GO**	87
**KEGG**	25
**COG**	35
**Total**	90

Annotation of the 90 candidate genes contributed to the further study of map-based gene isolation. The details about 90 candidate genes annotation information from GO, COG and KEGG databases were showed in S1, S2 and S3 Figs. From genetic and molecular based research on rice yield, it is known that grain weight is controlled by cell division in the outer glumes and the grain filling rate [43]. For example, in rice, the genes of GS3 and qGL3 negatively regulate cell division in the outer glumes so that the loss of their functions increase grain yield [44–46]. Previous studies on rice and *Arabidopsis* concluded that IAA might play an important role in regulating cell number. For sink organs of rice, the *tgw6* allele affects the timing of the transition from the syncytial to the cellular phase by controlling IAA supply and limiting cell number and grain length [47, 48]. From the annotated information in our study, we found four interesting candidate genes, GSBRNA2T00037136001, GSBRNA2T00037157001, GSBRNA2T00037129001 and GSBRNA2T00069389001. GSBRNA2T00037136001 participates in cell division; GSBRNA2T00037157001 was involved in the process of seed development; GSBRNA2T00037129001 was involved in both seed development and cell division; and GSBRNA2T00069389001 participated in the process of IAA biosynthesis, all of which might be highly related to seed weight.

## Conclusions

In this study, SLAF-seq technology combined with BSA was firstly and successfully used to detect candidate genes for seed weight in *B*. *napus*. A hot-region ~0.58 Mb with 91 candidate genes on ChrA09 were identified to be tightly associated with the TSW trait. The four most likely candidate genes were selected from annotation information. Confirmation of the function of these candidate genes by transformation or assessment of mutation for gene mining represents worthwhile future studies.

## Supporting Information

S1 Fig GO function classification diagram of 87 candidate genes in associated region according to cellular component, molecular function and biological process. (TIF)

S2 Fig COG function classification diagram of 35 association regional candidate genes. In different functional classes, the proportion of genes reflects the metabolic and physiological bias in corresponding period and environment.
(TIF)

S3 Fig An example of KEGG pathway for Glycolysis/ Gluconeogenesis (ko00010) of 25 association regional candidate genes. The number in the blue box represents the number of associated enzyme.
(TIF)

S1 Table The mean values of 1000-seed weight for three replicates of the DH population. (XLSX)

S2 Table Independent Sanger sequencing for quality validation of SLAF-seq genotyping (XLS)

S3 Table Annotation information for 91 candidate genes. (XLSX)
