# Supplementary figures and images for: Rapid Identification of Candidate Genes for Seed Weight Using the SLAF-Seq Method in Brassica napus
Source: PLoS One. 2016 Jan 29;11(1):e0147580. doi: 10.1371/journal.pone.0147580 (PMC4732658; doi:10.1371/journal.pone.0147580)

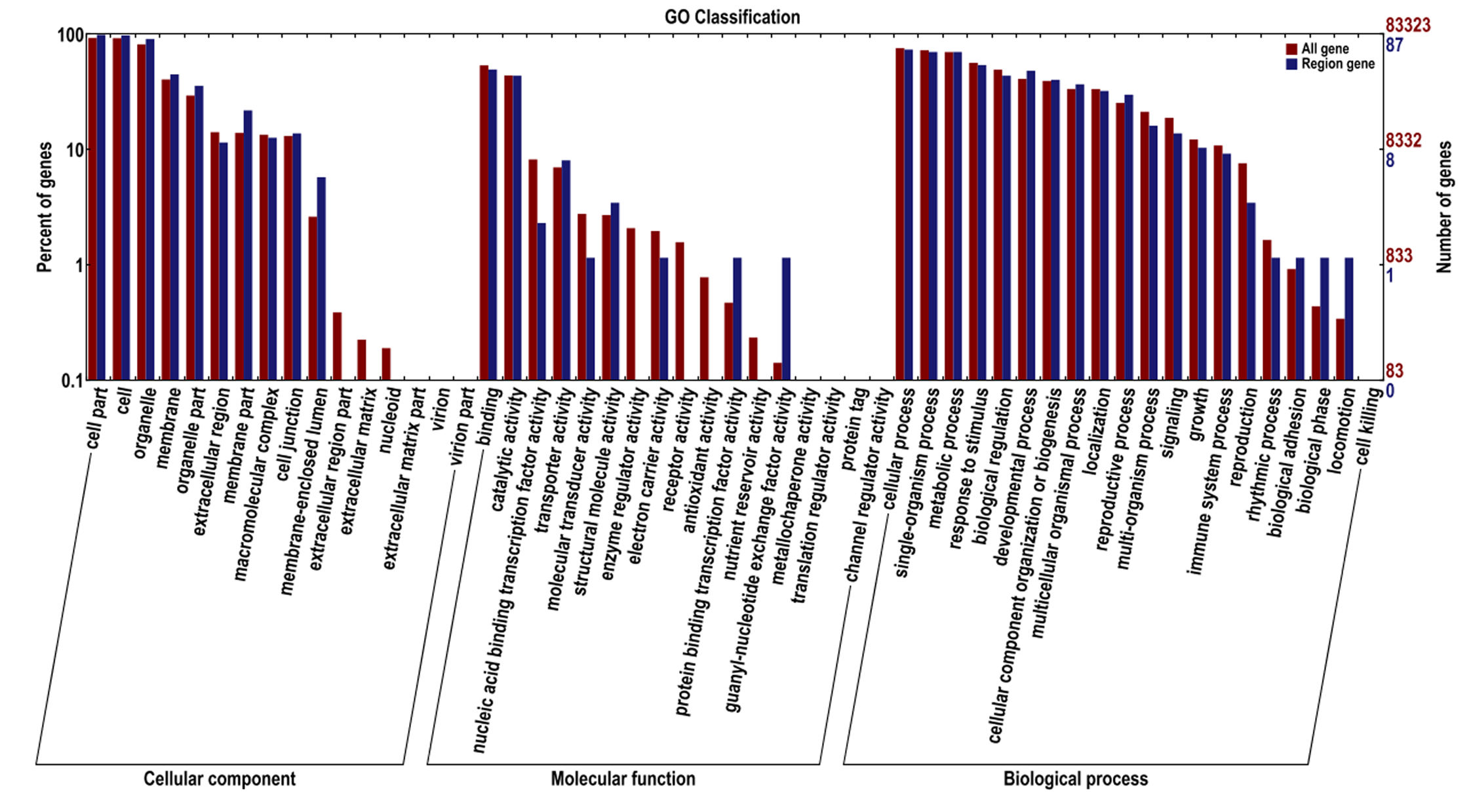

Supplement: S1 Fig — (TIF) [file pone.0147580.s001.tif]

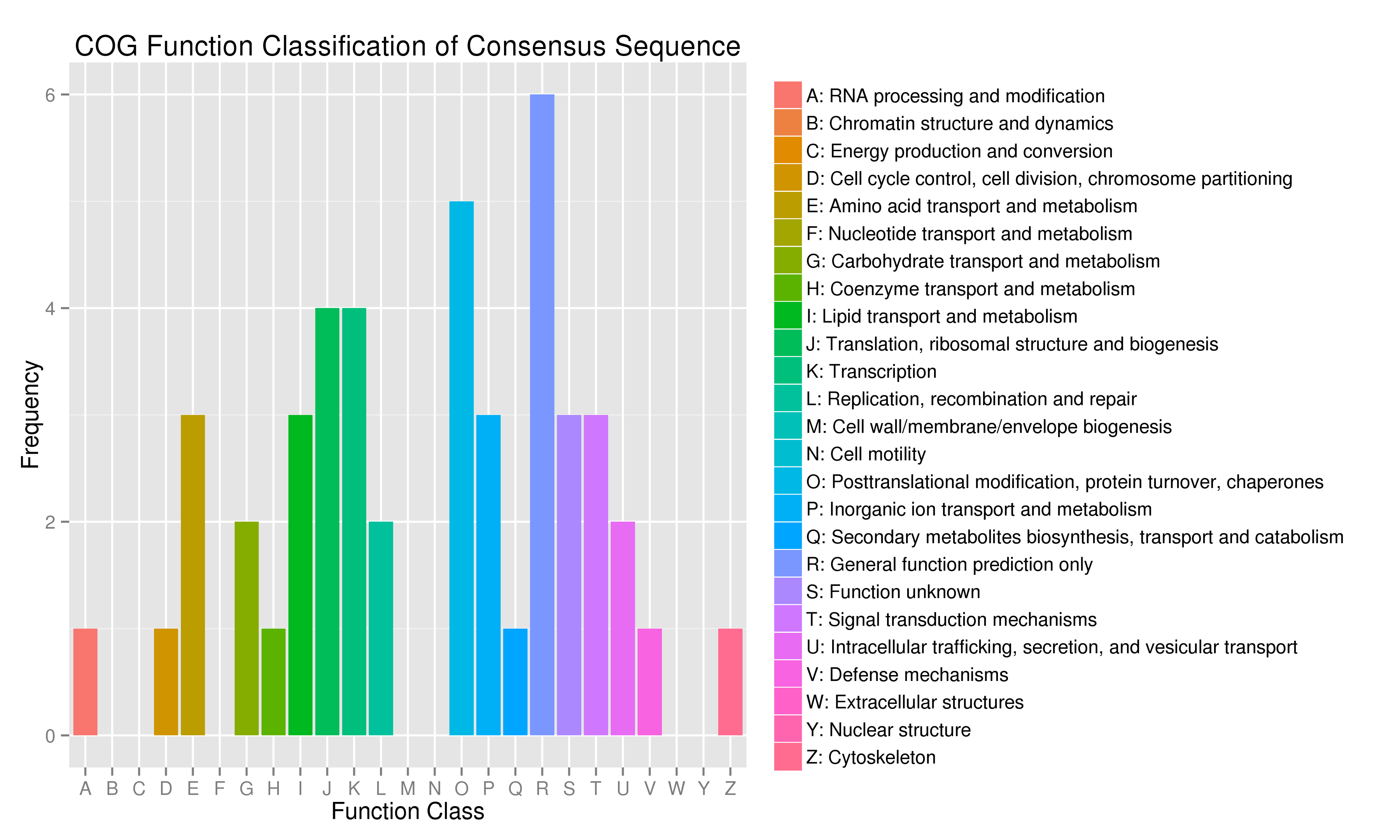

Supplement: S2 Fig — In different functional classes, the proportion of genes reflects the metabolic and physiological bias in corresponding period and environment. (TIF) [file pone.0147580.s002.tif]

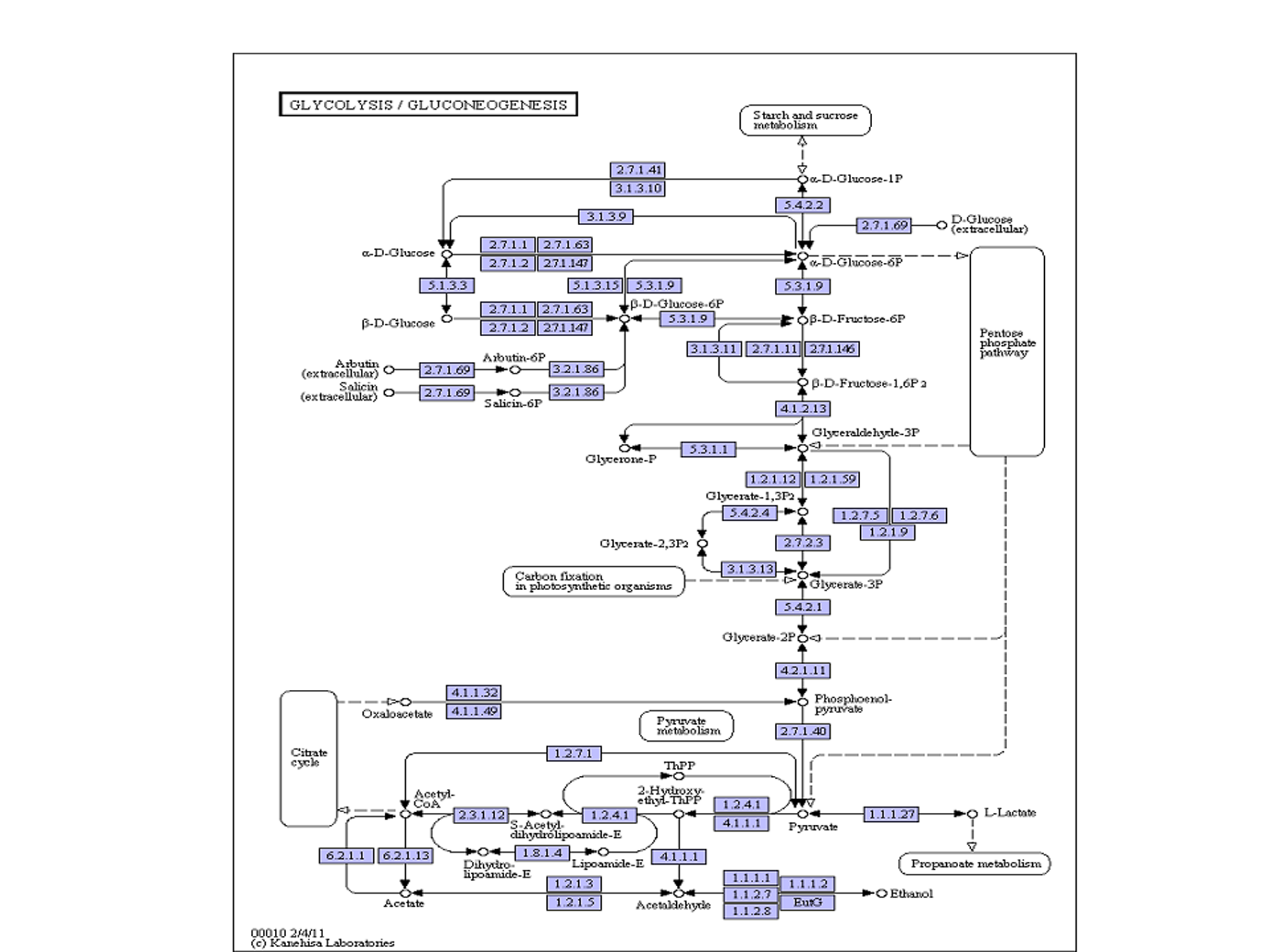

Supplement: S3 Fig — The number in the blue box represents the number of associated enzyme. (TIF) [file pone.0147580.s003.tif]
